# Incidence of metabolic bone disease in neonates under 32 gestational weeks at the Hospital Universitario de Santander in Colombia

**DOI:** 10.7705/biomedica.6926

**Published:** 2024-03-31

**Authors:** Erika Ruiz, Diego Ernesto Piamonte, Deisy Tatiana Gómez, Luis Alfonso Díaz, Luis Alfonso Pérez

**Affiliations:** 1 Escuela de Medicina, Facultad de Salud, Universidad Industrial de Santander, Bucaramanga, Colombia Universidad Industrial de Santander Escuela de Medicina Universidad Industrial de Santander Bucaramanga Colombia; 2 Departamento de Pediatría, Facultad de Salud, Universidad Industrial de Santander, Bucaramanga, Colombia Universidad Industrial de Santander Departamento de Pediatría Universidad Industrial de Santander Bucaramanga Colombia

**Keywords:** Bone diseases, metabolic, infant, premature, alkaline phosphatase, phosphorus, vitamin D, enfermedades óseas metabólicas, recién nacido prematuro, fosfatasa alcalina, fósforo, vitamina D

## Abstract

**Introduction.:**

Metabolic bone disease of premature infants is a rare complication characterized by a lower mineral content in bone tissue.

**Objective.:**

To establish the incidence of metabolic bone disease in premature infants and to determine associated risk factors.

**Materials and method.:**

We conducted a descriptive prospective cohort study for one year in all newborns under 32 gestational weeks, or 1,500 g, at the *Hospital Universitario de Santander* to determine the incidence of metabolic bone disease.

We collected demographic data and prenatal histories of the selected patients, and later, we measured serum alkaline phosphatase and serum phosphorus at the third week of birth, having as reference values for diagnosis less than 5.6 mg/dl for the first one and more than 500 UI/L for the second one.

We applied statistical tools for data analysis, such as average proportions, dispersion, distribution and association measures, and binomial regression.

**Results.:**

From a total of 58 patients, 7 had a diagnosis of metabolic bone disease, with an incidence of 12%. The weight was reported as an independent variable for the development of the disease, being significant in children under 1,160 g, as well as prolonged parenteral nutrition for more than 24 days. When performing the multivariate analysis, low weight and short time of parenteral nutrition appeared as risk factors; in the same way, maternal age below 22 years is associated with a higher relative risk, even more than a newborn weight inferior to 1,160 g.

**Conclusion.:**

Establishing an early intervention in patients with metabolic bone disease enhancing risk factors, such as low weight and prolonged parenteral nutrition, is critical to prevent severe complications.

## Resumen

**Introducción.:**

La enfermedad metabólica ósea de neonatos prematuros es una complicación poco común que se caracteriza por una disminución del contenido mineral en el hueso.

**Objetivo.:**

Establecer la incidencia de la enfermedad metabólica ósea en neonatos prematuros y los factores de riesgo asociados.

**Materiales y métodos.:**

Durante un año, se realizó un estudio prospectivo de cohorte, descriptivo, con todos los neonatos nacidos con menos de 32 semanas de gestación o un peso menor de 1.500 g en el Hospital Universitario de Santander.

Se recolectaron datos demográficos y antecedentes prenatales de los pacientes seleccionados. A la tercera semana de nacimiento, se midieron la fosfatasa alcalina y el fósforo sérico, tomando como valores de referencia diagnóstica aquellos inferiores a 5,6 mg/dl para el primero y aquellos mayores de 500 UI/L para la segunda.

Para el análisis de la información, se emplearon herramientas estadísticas, como proporciones de promedios, medidas de dispersión, distribución y asociación, y regresión binomial.

**Resultados.:**

De un total de 58 pacientes, 7 tuvieron diagnóstico de enfermedad metabólica ósea, con una incidencia del 12 %. De las variables estudiadas, el peso se reportó como una variable independiente para el desarrollo de la enfermedad, significativa en aquellos neonatos con peso menor de 1.160 g, al igual que la nutrición parenteral prolongada por más de 24 días. Al hacer el análisis multivariado, La edad materna menor de 22 años representó un riesgo relativo mayor, en comparación con un peso inferior a 1.160 g.

**Conclusión.:**

Se estableció la importancia de una intervención temprana en pacientes con factores de riesgo para enfermedad metabólica ósea, como bajo peso (menor de 1.160 g) y nutrición parenteral prolongada (mayor de 24 días), con el fin de prevenir complicaciones graves.

Metabolic bone disease of preterm infants is a medical condition preceded by inadequate aggregation of the bone mineral component with biochemical downregulation of calcium and phosphorus, insufficient in neonates of younger gestational age ^1^^,^^2^.

Although the incidence remains unknown, a 60% occurrence has been reported in preterm infants weighing less than 1,000 g, with fracture rates of 2% to 10%, and affecting 23% of newborns weighing less than 1,500 g ^3^.

In fetal life, the fetus receives 80% of the micronutrients by the 24^th^ week. At birth, the calcium passage is interrupted, and levels drop suddenly in neonates weighing between 500 and 2,000 g. Calcium requirements vary from 99 to 173 mg/kg/day, and phosphorus, from 63 to 126 mg/kg/day ^2^^,^^4^.

To develop the disease, rapid depletion of substrates, especially phosphorus, must occur due to urinary loss caused by the parathyroid hormone. Exclusive breastfeeding and poor supplementation also result in lower phosphorus stores, while vitamin D increases intestinal absorption. Nutrient supplementation in preterm infants is nearly 60% of calcium and 80 90% of phosphorus ^5^.

Clinical manifestations appear between the 3^rd^ and the 12^th^ week after birth, but they can be asymptomatic for several weeks. The severity of metabolic bone disease is directly related to the increase in alkaline phosphatase levels and the decrease in serum phosphorus, reference serum markers to establish the diagnosis. Alkaline phosphatase levels below 500 IU/L determine normal bone density. Calcium levels affect bone densitometry, the method of choice to assess bone mineralization, because the results are independent of anthropometry and gestational age. However, bone densitometry availability is scarce ^6^.

Regarding clinical presentation, symptoms can be as severe as pathological rib fractures in up to 32% of patients, respiratory distress, myopia, and long-term low bone mineral content during growth, implying an increased risk of osteoporosis.

Management is based on the supplementation of calcium carbonate, vitamin D, and phosphorus to meet the requirements of neonates with metabolic bone disease, from doses established in the international consensus: 10-20 mg/kg/day with a maximum dose of 40-50 mg/kg/day for phosphorus, 20 mg/kg/day with a maximum dose of 40-50 mg/kg/day for calcium carbonate, and 400-1,000 IU/day for vitamin D, the major factor to reduce the incidence of metabolic bone disease ^7^^,^^8^.

Biochemical follow-up will depend on the disease severity and the clinical context. Medical staff can gradually decrease mineral supplements to avoid adverse reactions as the markers approach their normal state. Clinical practice guidelines recommend measuring alkaline phosphatase two to four weeks after discharge in exclusively breastfed very lowbirth-weight infants with direct mineral supplements if alkaline phosphatase is over 800 to 1,000 IU/L. Closer follow-up is required in those patients with a moderate to severe condition or with a constant risk of deficient bone mineralization since they may require prolonged fortification or direct mineral supplementation. The latter should be monitored with biochemical markers ^8^.

In metabolic bone disease, population characterization and diagnostic cut-off points through serum markers are essential since metabolic bone disease has an unfavorable impact in childhood, such as a reduction in bone mass and low height compared to the reference population ^9^. For this reason, we conducted a study to establish the incidence of metabolic bone disease and to determine associated risk factors, including medications like caffeine and steroids.

## Materials and methods

For one year, we conducted a descriptive, prospective cohort study of preterm newborns hospitalized at the *Hospital Universitario de Santander*.

All newborns with less than 32 gestational weeks, without discrimination of underlying pathologies or neonatal evolution, were included; and only those that remained hospitalized for less than three weeks, due to discharge, transfer, or death, were excluded.

We collected demographic and perinatal data, clinical evolution, including intrauterine growth restriction, and the use of methylxanthines to describe associated risk factors (table 1). The data were collected in a form established and approved by the ethics committee after the mother or legal guardian of the admitted newborn signed the informed consent. Later, we transferred the data to the database created in the REDCap system, complying with all safety criteria according to the current ethical standards.

**Table 1:** Considered variables in the study

Prenatal history	Immediate neonatal		Neonatal evolution	
a. Preeclampsia	a. Sex		a. Airway support	
b. Intrauterine Growth restriction	b. Weight		b. Hemodynamic support	
c. Chorioamnionitis	c. Mass body index		c. Use of:	
d. Gestational diabetes or pre-diabetes		• Gestational age (obstetric data: first trimestrer ultrasound, last menstrual period, physical exam)		• Diuretics
e. Thyroid disease				• Methylxanthines
f. Prenatal corticosteroid use (two or more doses)	d. Basic or advanced life support		d. Parenteral nutrition over four weeks	
g. Unique or multiple pregnancy	e. Oxygen requirement		e. Presence of:	
	f. Continuous positive airway pressure			• Bronchopulmonary dysplasia
	g. Compressions			• Sepsis
	h. Vasopressors			• Necrotizing enterocolitis

The diagnosis of metabolic bone disease was established using serum alkaline phosphatase and phosphorus reference values (table 2). We took neonates’ blood samples at the third week after birth or earlier if there was clinical suspicion (tenderness on bone palpation or bone deformities on physical examination) or incidentally found fractures.

**Table 2:** Reference values for phosphorus and alkaline phosphatase

	^Reference^	^Metabolic bone disease^
Phosphorus	> 1.81 mmol/L (5.6 mg/dl) to 2.91 mmol/L (9.01 mg/dl)	< 1.81 mmol/L (5.6 mg/dl)
Alkaline phosphatase	< 500 UI/L	> 500 UI/L

Patients diagnosed with metabolic bone disease received ionic supplementation at doses ranging from 10 -20 mg/k/day to a maximum of 40-50 mg/kg/day for calcium carbonate and 400-1000 IU/day for vitamin D, according to the international consensus. The clinical practice guidelines for parenteral and enteral nutrition at the *Hospital Universitario de Santande*r already included phosphorus supplementation.

We weekly monitored diagnosed patients based on alkaline phosphatase and phosphorus laboratory results until they reached normal levels and thus discontinued supplementation. In addition, endocrinology follow-up was indicated at hospital discharge.

### Statistical analysis

We determined nominal and ordinal proportions, such as mean and standard deviation or median and interquartile range for discrete or continuous variables, whether or not they had a normal distribution. We then compared the incidence of metabolic bone disease among patients with a history of intrauterine growth restriction versus those without the condition and between those who received or did not methylxanthines. The differences found were evaluated using the x^2^test, with statistically significant differences defined as p<0.05. Finally, potential associations were assessed using binomial regression adjusted for confounding bias due to the incidence of infections, among others.

### Ethics

This research work meets the requirements of the national and international regulations. According to Resolution 008430 of October 4^th^, 1993, this study has minimal risk, with clinical procedures consisting of a routine diagnostic test carried out through venous puncture in neonates. The extracted volume represents less than 2% of the neonate’s total blood.

Metabolic bone disease is a condition typical of the neonatal period, of scientific interest, and of great value to those suffering it, with the screening and diagnostic mechanisms we described here. According to chapters 15 and 16 of the above mentioned resolution, our research has the duly completed informed consent, signed by the mother or legal guardian of the patient, and given prior information on the risks and benefits.

The participants benefited from the screening carried out worldwide to diagnose metabolic bone disease and appropriate disease management in the medium and long term. In the same way, we will have the possibility for the routine determination of a little-known disease in our context.

The project was reviewed by the *Comité de Ética en Investigación Científica* (*CEINCI*) at the *Universidad Industrial de Santander* and the *Comité de ética* of the *Hospital Universitario de Santander*.

## Results

**Figure 1:**
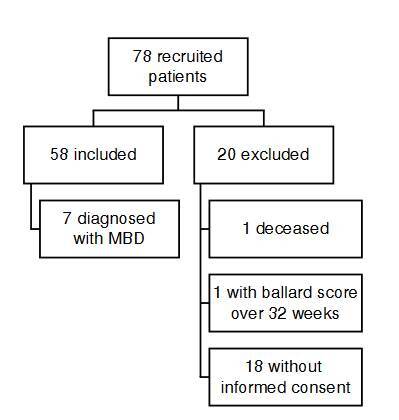
Inclusion and exclusion

We compiled samples from 78 newborns admitted to the Hospital Universitario de Santander neonatal unit between 2020 and 2021. Fifty eight patients met the inclusion criteria, and 20 were excluded (figure 1): 18 patients did not have informed consent, one died, and one had a Ballard score over 32 weeks.

MBD: metabolic bone disease

The demographic characteristics of the global sample contemplates maternal and neonatal populations (table 3). We found a predominance of the female sex (55.1%) compared to the male sex (44%). The studied variables are described in table 4. Within the representative data, supplemental oxygen was used in all patients as the first step in neonatal resuscitation, followed by the INSURE maneuver in 98.2%.

**Table 3:** Neonatal and maternal demographic characteristics

Variables		Total n (%)	
Sex [n (%)]			
	Male	26	(44.8)
	Female	32	(55.1)
Median of gestational age [n (IQ)]		30	(28-31)
Way of delivery [n (%)]			
	Cesarean section	46	(79.3)
	Vaginal delivery	12	(20.7)
Birth weight [g (IQ)]		1,334.7	(600-2,005)
Average birth weight [g (IQ)]		1,350	(1,185-1,540)
Prenatal steroid use [n (%)]		40	(71.4)
Neonatal life support [n (%)]			
	Oxygen	58	(100)
	INSURE maneuver	57	(98.2)
	CPAP	56	(96.5)
	Thoracic compressions	7	(12.0)
	Vasopressors	7	(12.0)
Neonatal intensive care unit requirement		39	
Morbidities [n (%)]			
	Bronchopulmonary dysplasia	23	(39.6)
	Necrotizing enterocolitis	12	(20.6)
	Neonatal sepsis	33	(56.9)
Pharmacological exposure [n (%)]			
	Caffeine citrate	49	(84.4)
	Furosemide	12	(20.7)
	Corticosteroids	21	(36.2)
	Parenteral nutrition	51	(87.9)
Metabolic bone disease diagnosis [n (%)]			
	Yes	7	(12.0)
	No	51	(88.0)
Maternal characteristics		26	(22-31)
Median of maternal age - n (IQ)			
Comorbidities [n (%)]			
	Preeclampsia	21	(37.5)
	Chorioamnionitis	18	(32.1)
	Intrauterine growth restriction	4	(7.1)
	Hypothyroidism	0	(0.0)

INSURE: Intubation-Surfactant-Extubation; CPAP: Continuous Positive Airway Pressure; NICU: Neonatal Intensive Care Unit; MBD: Metabolic Bone Disease; IQ: Interquartile Range

**Table 4:** Factors associated with metabolic bone disease

		MBD (N=7)	Non-MBD (N=51)	p value
Maternal age (years)				
	< 21	4	7	0.006
	> 22	3	44	
Maternal comorbidities				
	Preeclampsia	3	18 (35.2%)	0.696
	Gestational diabetes	1	5 (13.1%)	0.678
	Chorioamnionitis	2	16 (31.3%)	0.881
Prenatal corticosteroids		4	37 (78,7%)	0.506
Gestational age (weeks)				
	< 28	3	9	0.010
	> 28	4	49	
Sex				0.080
	Female	6	32	
	Male	1	26	
Average birth weight [g (range)]		980 (674-1,455)	1,375 (600-2,005)	0.014
	< 1,000	4	7	
	1,000-1,500	3	28	
	> 1,500	0	16	
Apgar score < 3 points		3	9 (17.65%)	
Neonatal reanimation				0.123
	Oxygen	7	50 (98%)	-
	INSURE	7	50 (98%)	0.789
	CPAP	7	49 (96%)	0.594
	Thoracic compressions	2	5 (9.8%)	0.153
	Mechanical ventilation	2	5 (9.8%)	0.153
	NICU	4	35 (68.6%)	0.544
Neonatal comorbidities				
	Bronchopulmonary dysplasia	4	19 (37.2%)	0.313
	Necrotizing enterocolitis	4	8 (15.6%)	0.011
	Neonatal sepsis	7	26 (50.9%	0.014
Pharmacological intervention				
Caffeine citrate [days of use]		7	42 (82.3%)	0.227
	< 28	0	3	0.275
	> 28	7	39	0.677
Furosemide [days of use]		2	11 (21.5%)	
	< 5	1	8	0.338
	5-10	0	3	
	> 10	1	0	
Postnatal corticosteroids [days of use]		4	17(33.3%)	0.219
	< 7	3	15	0.195
	7-14	0	0	
	> 15	1	2	
Nutrition				
Parenteral nutrition		7	44 (86.2%)	0.296
Days of use [median) (IQ)]		37	13 (0-60)	0.002
	< 7	0	9	
	7-14	0	18	
	15-30	4	14	
	> 30	3	3	
Exclusive breastfeeding		0	51 (100%)	-
Mixed breastfeeding		7	47 (92.1%)	0.443
Fortified milk		2	6 (11.7%)	0.227

INSURE: Intubation-Surfactant-Extubation; CPAP: Continuous positive airway pressure; NICU: Neonatal intensive care unit; MBD: Metabolic bone disease; IQ= Interquartile range

The main neonatal morbidity was sepsis in 56.9%, followed by bronchopulmonary dysplasia in 39.6% and necrotizing enterocolitis in 20.6%. Eighty-four-point four percent received caffeine citrate for the premature condition.

### 1. Univariate analysis of factors associated with metabolic bone disease

The incidence of metabolic bone disease was 12.0%, mostly with a gestational age of less than 28 weeks (p=0.020), and predominance of female sex (85.7% of the cases). Within the maternal morbidities prevailed preeclampsia, followed by chorioamnionitis and gestational diabetes. However, we did not find a statistically significant association.

All patients with metabolic bone disease had neonatal sepsis in common, necrotizing enterocolitis (57.4%), and long parenteral nutrition (average of 37 days), the three of them with statistical significance. From the latter, four patients received parenteral nutrition between 15 and 30 days and three patients for more than 30 days.

No link was found with the use of caffeine citrate, diuretics, postnatal steroids, or oral nutrition. No link was observed with the requirement of neonatal resuscitation or admission to intensive care.

### 2. Multivariate analysis

The multivariate analysis of the significant variables involved in metabolic bone disease development (table 5) revealed that parenteral nutrition over 24 days, maternal age less than 22 years, low birth weight, and comorbidities -such as enterocolitis and neonatal sepsis- appeared as association factors. The analysis indicates that parenteral nutrition for more than 24 days is an independent variable related to the development of metabolic bone disease (RR=18.85; CI 95% 2.47-143.5; p=0.005).

**Table 5:** Representative variables associated with metabolic bone disease

Variable	Relative risk	Standard error	p value	IC 95%
Parenteral nutrition (> 24 days)	7.95	8.25	0.04	1.04-60.72
Maternal age (< 22 years)	4.18	2.15	0.01	1.52-11.48
Corticosteroids	0.92	0.00	0.00	0.91-0.91
Birth weight (< 1,160 g)	3.19	0.01	0.08	0.85-11.91

Birth weight less than 1,160 g also has an independent relation with metabolic bone disease (RR=9.58; CI 95%=2.11-43.45; p=0.003).

Although necrotizing enterocolitis had a significant association, when including the parenteral nutrition variable, this link disappeared. Steroid use had no independent relation, but when including parenteral nutrition, maternal age, and birth weight less than 1,160 g, steroids show a protective effect with a significant pvalue.

## Discussion

Currently, the care of preterm newborns has been improved, along with prevention, early detection, and progression of age-related diseases. The scientific and clinical communities have an open interest in less prevalent diseases like metabolic bone disease, which is the object of our study.

The global reported incidence of metabolic bone disease is between 16 40%, and a recent study informed an incidence of 12.3%, similar to our study (12%; n=7) ^3^^,^^10^. The metabolic bone disease studied population had a statistical significance associated with gestational age, birth weight, and time of parenteral nutrition. This finding is supported by prospective studies and meta-analysis relating birth weight, parenteral nutrition time, and prolonged use of diuretics with metabolic bone disease development ^10^^-^^12^.

On the other hand, Chen *et al*. recently examined metabolic bone disease risk factors in an observational cohort study of 16 newborns diagnosed with metabolic bone disease. Using logistic regression analysis, the authors showed that gestational age under 30 weeks and achievement of full enteral nutrition beyond 28 days are independent risk factors ^13^.

Although these studies have many differences, we can establish common risk factors like low birth weight and parenteral nutrition for more than 24 days ^14^. In agreement with other studies, weight is an independent variable in metabolic bone disease development. This effect may be related to the pathophysiological mechanism of the disease since mineral deficiency in bone metabolism increases the disease risk by 9.5 times in children under 1,160 g (p=0.003; CI 95%=2.11-43.45).

In addition, maternal age less than 22 years old predisposes to preterm birth and intrauterine growth restriction, increasing the risk of developing metabolic bone disease.

Studies reporting caffeine citrate use and metabolic bone disease described an average treatment of 60 ± 45.8 days and an accumulated dose of 425.3 ± 235.2 mg. However, in our study, the median treatment time was 30 days, and the cumulative dose was 255 mg, values below the average reported in the literature showing caffeine citrate treatment as a non-significant variable. We could infer that the link between caffeine citrate and metabolic bone disease development depends on the treatment time. However, more multicenter studies with larger populations would be necessary to reach this conclusion.

Diuretic use such as furosemide has been linked to the pathophysiology of metabolic bone disease, explained by renal excretion of calcium. However, we did not observe a significant association. This effect may be attributed to the medication time of use, limited to less than five days, establishing a lower accumulated dose ^15^.

When evaluating prolonged parenteral nutrition over 24 days, we identified the impact on delaying the start of fortified enteral nutrition to prevent metabolic bone disease development. For this reason, it is essential to stabilize other comorbidities associated with prematurity. We must seek a balance between the start of early enteral nutrition and the progressive withdrawal of parenteral nutrition to allow an adequate supply of phosphorus and calcium ^16^.

Although we found a significant individual link between metabolic bone disease and necrotizing enterocolitis, when analyzed with parenteral nutrition, this association disappeared probably because the limitation of enteral feeding early initiation.

Neonatal sepsis is a risk factor for metabolic bone disease. However, some reports described no significant changes corresponding to a biochemical mechanism of reduced bone turnover ^17^.

The obtained results show the risk factors associated with metabolic bone disease development. Low birth weight and parenteral nutrition have the highest impact. Disease detection in its early stages is crucial to provide timely treatment and to avoid late complications. Despite not having a consensus for the diagnosis, serum biomarkers suit upon the disease pathophysiology, being useful for screening and diagnosis of metabolic bone disease.

Weight and prolonged parenteral nutrition are the most relevant risk factors to intervene in metabolic bone disease suffering population and could be used as screen markers for this disease in neonatal care centers. Follow-up of these risk factors will allow the implementation of early nutritional interventions in diagnosed patients, preventing severe complications.

Some of the limitations of our study are the population size, the lack of parathyroid hormone measurements, and the absence of metabolic bone disease diagnostic image interpretation. However, we found a correlation between p-values of phosphorus levels less than 5.6 mg/dL and alkaline phosphatase levels greater than 500 IU/L, used as cut-off points in the study.
